# Distinct mucosa-associated microbiota signatures and dysbiosis in Peutz-Jeghers syndrome polyps versus paired normal mucosa

**DOI:** 10.3389/fimmu.2026.1817311

**Published:** 2026-06-22

**Authors:** Shuai Tang, Lei Wang, Chong-xi Fan, Zhe-yi Han, Lei Zhang, Bai-rong Li, Shou-Bin Ning

**Affiliations:** Department of Gastroenterology, Air Force Medical Center, Beijing, China

**Keywords:** 16S rRNA gene sequencing, dysbiosis, mucosa-associated microbiota, Peutz-Jeghers syndrome, polyp pathogenesis

## Abstract

**Background:**

Peutz-Jeghers syndrome (PJS) is an *STK11/LKB1*-mutated autosomal dominant disorder with gastrointestinal hamartomatous polyps and elevated cancer risk, but the role of mucosa-associated microbiota in PJS polyp pathogenesis remains unclear.

**Aim:**

To investigate the mucosal microbial signatures, dysbiosis characteristics, and potential biomarkers in PJS polyps versus paired normal small intestinal mucosa.

**Methods:**

A paired-sample design was adopted, enrolling 44 genetically confirmed PJS patients. Mucosa-associated microbiota was profiled via 5R 16S rRNA gene sequencing. Statistical analyses included Wilcoxon rank-sum test, ANOSIM, Spearman’s correlation, and random forest modeling.

**Results:**

Alpha diversity (Observed species, Chao1, Shannon) was markedly reduced in polyps (all P < 0.001), while Simpson index increased (P < 0.001). Beta diversity differed significantly between groups (ANOSIM, R = 0.0667, P = 0.002). Polyps were enriched in *Pseudomonadota* (70.00% vs. 49.51%, P < 0.001) and depleted in *Bacillota*, *Bacteroidota* (both P < 0.001). Microbial dysbiosis index (MDI) was higher in polyps (P < 0.001). A 9-genera random forest model achieved AUC = 0.897. *Escherichia-Shigella* correlated with polyp number (P < 0.05), and *Enterococcus* correlated with CA72–4 levels (P < 0.05).

**Conclusion:**

PJS polyps exhibit significant mucosal microbial dysbiosis, which may serve as potential biomarkers and therapeutic targets for PJS.

## Introduction

Peutz-Jeghers syndrome (PJS) is an autosomal dominant disorder predominantly caused by mutations in the *STK11/LKB1* gene, clinically characterized by mucocutaneous pigmentation and gastrointestinal hamartomatous polyps. It has an estimated global incidence of 1 in 200,000 to 1 in 100,000 live births (1, 2). These lesions significantly increase the risk of multiple malignancies (1, 2), yet the mechanisms linked to polyp initiation, growth, and progression remain incompletely elucidated. Beyond germline genetics, mounting evidence underscores the intestinal microenvironment as a pivotal regulator of gastrointestinal homeostasis and disease pathogenesis (3).

The gut microbiota, particularly mucosa-associated microbial communities at the epithelial interface, modulates host immune responses, epithelial barrier function, and metabolic processes (4). Dysbiosis, defined as disruption in microbial composition and function, has been implicated in inflammatory bowel disease (IBD) and colorectal cancer (CRC) (5, 6). In sporadic CRC, specific microbial signatures are associated with adenomatous polyps and carcinomas, suggesting active contributions to tumorigenesis (7, 8). However, previous PJS microbiome studies relied primarily on fecal samples, which poorly reflect the mucosal surface microbiota (9). Fecal samples represent luminal microbes, whereas mucosa-associated communities directly interact with epithelial cells and immune cells at the polyp origin site, making them more relevant to polyp pathogenesis (10).

Notably, the mucosa-associated microbiota of Peutz-Jeghers syndrome (PJS) polyps relative to normal mucosa remains incompletely characterized. We hypothesize that the unique pathological microenvironment of PJS polyps is associated with localized microbial dysbiosis, with dysregulated host-microbe crosstalk modulating polyp initiation and growth. To fill this knowledge gap, we adopted a rigorous paired-sample design—matching polyp and normal small intestinal mucosa from the same patients—to minimize inter-individual variability. Using 5R 16S rRNA gene sequencing and advanced bioinformatics, we sought to compare microbial diversity and community structure between PJS polyps and normal mucosa, identify differentially abundant taxa and potential microbial biomarkers, predict functional alterations in the polyp-associated microbiota, and explore correlations between microbial composition and clinical parameters. This study offers foundational insights into the microecological dynamics of PJS and informs potential microbiota-targeted interventions.

## Materials and methods

### Participant recruitment and sample collection

A total of 44 patients with genetically confirmed PJS were recruited from the department of gastroenterology, Air Force Medical Center, between November 2024 and June 2025. All participants underwent transanal double-balloon enteroscopy (DBE) with polypectomy. All DBE procedures and polypectomies were performed by three senior gastroenterologists with ≥10 years of experience in small bowel endoscopy (>200 procedures/year). The procedure protocol was standardized across all operators. For each patient, ileal polyp tissues were excised from the central region of the lesion, avoiding contact with the surrounding luminal surface, and paired normal mucosal samples were collected from the terminal ileum at least 10 cm proximal to the site of polyp excision. All samples were placed in sterile cryovials within 30 seconds of collection and immediately snap-frozen in liquid nitrogen. Immediately after collection, each specimen was divided into two aliquots under aseptic conditions: one was snap-frozen in liquid nitrogen and stored at -80 °C for microbial DNA analysis, and the other was fixed in 10% neutral-buffered formalin for immunohistochemical studies. All patients received identical standardized bowel preparation: a clear liquid diet for 24 hours pre-procedure, followed by oral administration of 2 L of polyethylene glycol (PEG) electrolyte solution 6 hours prior to DBE. No additional laxatives or enemas were used.

The study protocol was approved by the Ethics Committee of Air Force Medical Center (2023-77-PJ01). Written informed consent was obtained from all participants (guardians for minors aged <18 years).

PJS was diagnosed based on one of the following criteria (11) (1): Two or more histologically confirmed Peutz-Jeghers-type hamartomatous polyps in the gastrointestinal tract (2); Any number of Peutz-Jeghers polyps with a family history of PJS in a first-degree relative (3); Characteristic mucocutaneous pigmentation with a family history of PJS in a first-degree relative (4); Characteristic mucocutaneous pigmentation plus any number of Peutz-Jeghers polyps.

Eligible participants were aged 14–80 years, had a body mass index (BMI) < 30 kg/m² (any gender), and presented with stable vital signs. Exclusion criteria included significant comorbidities (e.g., cardiopulmonary, hepatic, or renal insufficiency), specific diagnoses (e.g., ascites, jaundice, cirrhosis, coagulation disorders), physiological states (e.g., pregnancy, breastfeeding), acute clinical conditions (e.g., fever, active gastrointestinal bleeding), a history of colorectal malignancy, contraindications to DBE or uncooperativeness, use of antibiotics, probiotics, prebiotics, laxatives, proton pump inhibitors (PPIs), non-steroidal anti-inflammatory drugs (NSAIDs), immunosuppressants, or corticosteroids within 2 weeks prior to sampling, and inability to provide complete clinical information.

All enrolled patients were of Han Chinese ethnicity. Detailed information on patients’ diet (past 3 months dietary patterns), lifestyle habits (smoking, alcohol consumption, physical activity), and medication history was collected via standardized questionnaires. All participants followed a traditional Chinese diet characterized by high carbohydrate intake (predominantly rice and wheat), moderate protein intake, and relatively low fat consumption. No participants reported following special dietary patterns (e.g., vegetarian, ketogenic, gluten-free) in the 3 months prior to sampling.

### DNA extraction and 5R 16S rRNA gene sequencing

Total microbial genomic DNA was extracted using the YHUltra-Trace DNA Extraction Kit (MJYH Bio-Pharm Technology Co., Ltd., Shanghai, China, Cat. No. MJYH-D8101), a kit specifically designed for ultra-low biomass samples, following the manufacturer’s standard protocol with minor modifications for intestinal mucosal tissues. DNA quality was verified by 1% agarose gel electrophoresis, while concentration and purity were determined using a NanoDrop 2000 spectrophotometer (Thermo Fisher Scientific, USA).

For microbial community profiling, 5R 16S rRNA gene sequencing was employed to amplify five hypervariable regions (V2, V3, V5, V6, V8) of the bacterial 16S rRNA gene, which enhances coverage and taxonomic resolution for low-biomass samples. Barcoded specific primers targeting these five regions were synthesized, with detailed sequences provided in **Table 1**. Multiplex PCR was performed on an ABI GeneAmp^®^ 9700 thermocycler (Thermo Fisher Scientific, USA). After pooling, PCR products were purified with the AxyPrep DNA Gel Extraction Kit (Axygen Biosciences, USA) and eluted in Tris-HCl buffer. Purified products were re-verified via 2% agarose gel electrophoresis, quantified using a Quantus™ Fluorometer (Promega, USA), and subjected to paired-end sequencing on an Illumina MiSeq PE300 platform (Illumina, USA) following Majorbio Bio-Pharm Technology Co., Ltd. (Shanghai, China) standard protocols. The raw 16S rRNA gene sequencing reads have been submitted to the NCBI Sequence Read Archive (SRA) and are accessible under the BioProject accession number PRJNA1402014.

**Table 1:** Primers used for 16S rRNA gene sequencing.

Sequencing region	Forward primer sequence (5′→3′)	Reverse primer sequence (5′→3′)
V2	F1-TGGCGAACGGGTGAGTAA	R1-CCGTGTCTCAGTCCCARTG
V3	F2-ACTCC TACCGGGAGGCAGC	R2-GTATTACCGCGGCTGCTG
V5	F3-GTGTAGCGGTGRAATGCG	R3-CCCGTCAATTCMTTTGAGTT
V6	F4-GGAGCATGTGGWTTAATTCGA	R4-CGTTGCGGGACTTAACCC
V8	F5-GGAGGAAGGTGGGGATGAC	R5-AAGGCCCGGGAACGTATT

The degeneracy codes in the primer sequences are: R (A or G), M (A or C), W (A or T), N (any base).

### Bioinformatic processing

All experimental procedures included appropriate negative controls to monitor for reagent and laboratory contamination, as detailed below. Sequences from the five targeted regions were integrated using the Short MUltiple Regions Framework (SMURF) pipeline. Taxonomic assignment was performed Greengenes 16S rRNA database (May 2013 version). To address contamination risks in ultra-low biomass mucosal samples, we implemented a two-step filtering pipeline (1): pre-filtering removed samples with <1000 reads and taxa <10^-4^ relative abundance (2); negative control filtering (5 extraction blanks + 5 PCR blanks) eliminated taxa present in ≥30% of either control set. Purified amplicon sequence variants (ASVs) were generated to characterize microbial profiles. Alpha diversity indices were calculated, including observed species (Sobs), Chao1 richness estimator, Shannon diversity index, and Simpson’s diversity index (1-D). This version of the Simpson index ranges from 0 to 1, where higher values indicate lower microbial diversity (i.e., greater dominance by a small number of taxa). Beta diversity was assessed using Bray-Curtis dissimilarity distances. Principal coordinate analysis (PCoA) and non-metric multidimensional scaling (NMDS) visualized intergroup structural differences, with statistical significance assessed via analysis of similarities (ANOSIM). The microbial dysbiosis index (MDI) was calculated as described by Gevers et al. (12). Overlapping ASVs between groups were illustrated using a Venn diagram (Python v2.7.10). Taxonomic abundance differences between polyp and normal mucosa samples were compared via the Wilcoxon rank-sum test. Linear discriminant analysis effect size (LEfSe) identified differentially abundant taxa (phylum to species level) with thresholds set at LDA score > 3.5 and P < 0.05. Similarity percentages (SIMPER) analysis determined the contribution of specific taxa to community dissimilarity.

### Microbiota function prediction and clinical correlation analysis

Microbiota functional profiles were predicted using PICRUSt2 (v2.2.0-b). Raw pathway counts were normalized to relative abundance by dividing each pathway’s count by the total pathway count per sample. Pathways with a mean relative abundance < 0.01% across all samples were filtered out to reduce noise. Spearman’s rank correlation coefficient analyzed associations between microbiota composition and clinical parameters, visualized via R (v3.3.1) and Python (v2.7). Polyp burden was evaluated using two metrics (1): polyp number (total endoscopically confirmed small bowel polyps) and (2) largest polyp diameter (maximum dimension of the largest identified polyp), reflecting disease extensiveness and local lesion severity, respectively. Polyp burden was measured by a single clinician (the chief endoscopist) for all procedures. For multi-polyp cases, the largest polyp was biopsied. Inter-observer variability was minimized by having a second independent reviewer verify the measurements.

### Statistical analysis

All statistical analyses were performed using R (v3.3.1), Python (v2.7), and SPSS (v27.0). Intergroup differences were tested via the two-sided Wilcoxon rank-sum test, with P-values corrected using the false discovery rate (FDR) method. Receiver operating characteristic (ROC) curve analysis evaluated the discriminatory power of microbial signatures. A two-sided P < 0.05 was considered statistically significant. Data processing was supported by the Majorbio Cloud Platform (https://www.majorbio.com).

## Results

### Clinical data

The clinical characteristics of the 44 patients (all Han Chinese ethnicity) are summarized in **Table 2**. The cohort included 30 males and 14 females, with a mean age of 30.00 ± 10.07 years. The mean body mass index (BMI) was 21.74 ± 3.62 kg/m². Previous surgical bowel interventions (excluding endoscopic polypectomy) were reported in 33 patients (75.00%). 5 patients had intestinal intussusception during this hospitalization (11.36%). Laboratory parameters at admission indicated anemia in 7 patients (15.91%). All patients underwent transanal DBE with polypectomy, and matched polyp and normal mucosal samples were collected as described.

**Table 2:** Clinical characteristics of 44 PJS patients.

Parameter	Value
Age (years), mean ± SD	30.00 ± 10.07
Male/Female	30/14
BMI (kg/m²), mean ± SD	21.74 ± 3.62
Ethnicity	All Han Chinese
Previous bowel surgery, n (%)	33 (75.00)
Small bowel resection	21
Ileocolic resection	7
Intussusception reduction	4
Anemia, n (%)	7 (15.91)

### Venn diagram analysis

**Figure 1:**
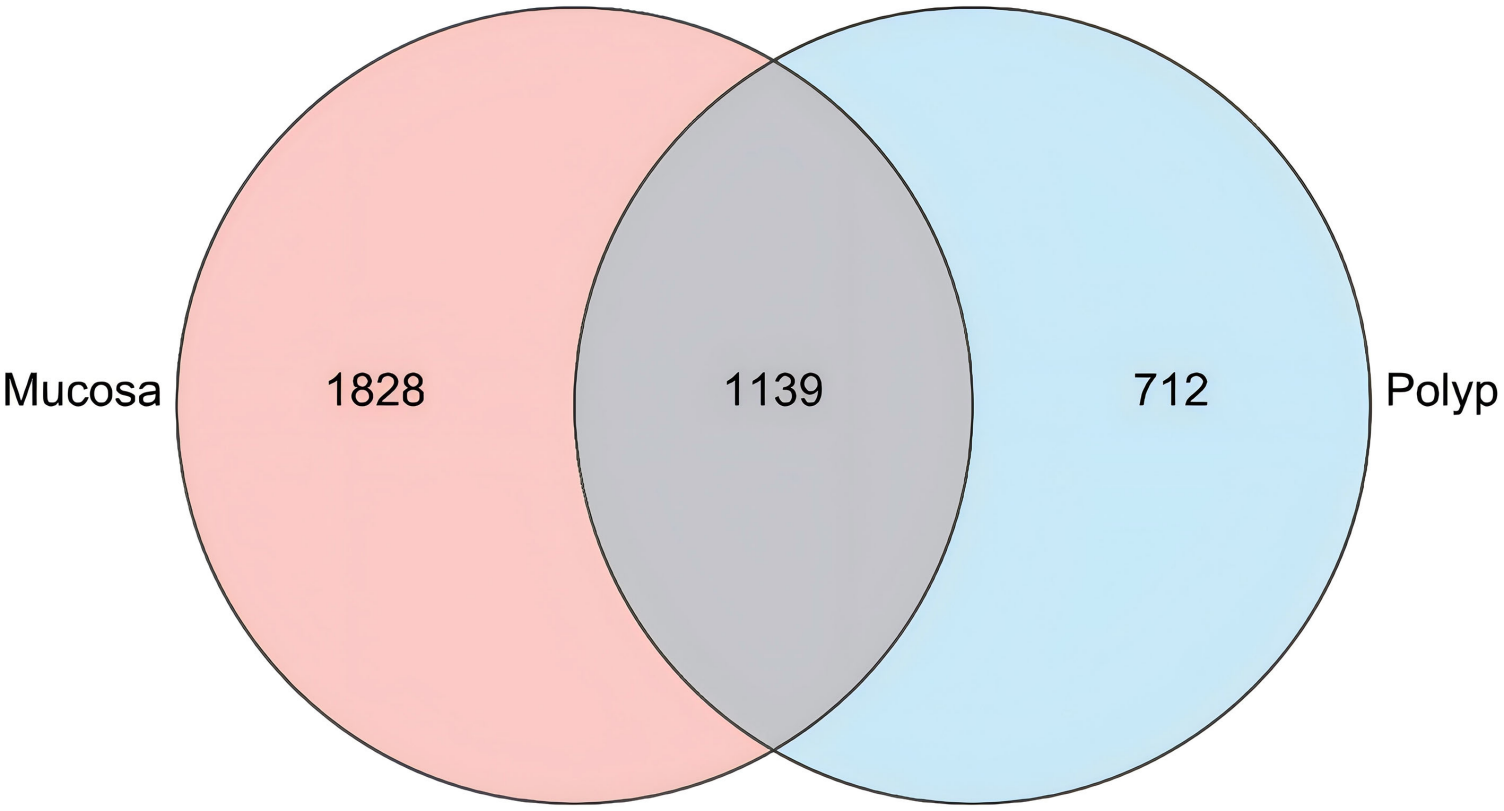
Species-level overlap of the mucosal microbiota between normal mucosa and polyp tissues. Venn diagram illustrates the number of shared and unique bacterial species between the mucosa (pink, n = 1828) and polyp (blue, n = 712) groups. The two groups shared 1139 common species.

A total of 19,711,824 high-quality sequences were obtained from 88 samples (44 pairs), with a mean of 223,998 sequences per sample. Taxonomic profiling identified 3,679 species, classified into 903 genera, 343 families, 120 orders, 54 classes, and 31 phyla. At the species level, the normal mucosa group contained 2,967 species (1,828 unique, accounting for 61.6% of the group’s total), while the polyp group contained 1,851 species (712 unique, accounting for 38.4%) (**Figure 1**). A total of 1,139 species were shared between the two groups, indicating substantial microbial overlap but a 60.9% reduction in unique taxa in polyps compared to normal mucosa.

### Alpha-diversity analysis

**Figure 2:**
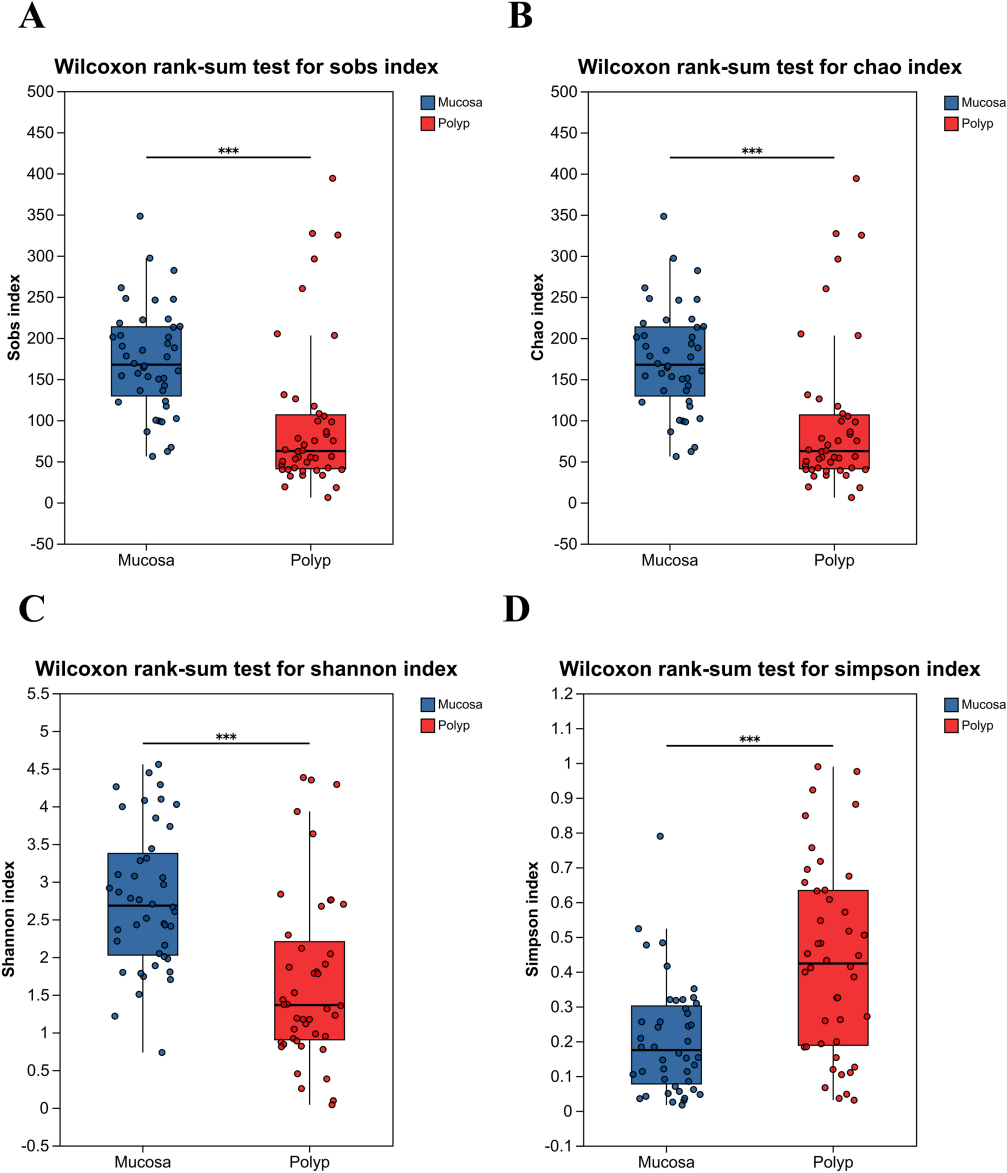
Comparison of alpha diversity of the mucosa-associated microbiota between normal mucosa and polyp tissues. Box plots showing the distribution of four alpha diversity indices: **(A)** observed species (Sobs), **(B)** Chao1, **(C)** Shannon, and **(D)** Simpson’s diversity index (1-D). The normal mucosa group (blue) and the polyp group (red) were compared using the Wilcoxon rank-sum test. *** indicated a significant difference at P < 0.001.

Mucosa-associated microbial alpha diversity was significantly reduced in PJS polyp tissues relative to paired normal mucosa. Four standard indices were applied, including Observed species (Sobs), Chao1, Shannon, and Simpson’s diversity index (1-D). The Sobs index was markedly decreased in polyps (p < 0.001), and the Chao1 index was also reduced (p < 0.001). The Shannon index was lower in polyps (p < 0.001), while Simpson’s diversity index (1-D) was significantly higher (p < 0.001). This index ranges from 0 to 1, with higher values indicating lower microbial diversity and greater taxonomic dominance, which is consistent with the decreasing trend observed in other alpha diversity metrics. These findings collectively confirm that PJS polyps harbor an impoverished, less diverse mucosal microbiota compared with normal mucosa (**Figure 2**).

### Beta-diversity analysis

**Figure 3:**
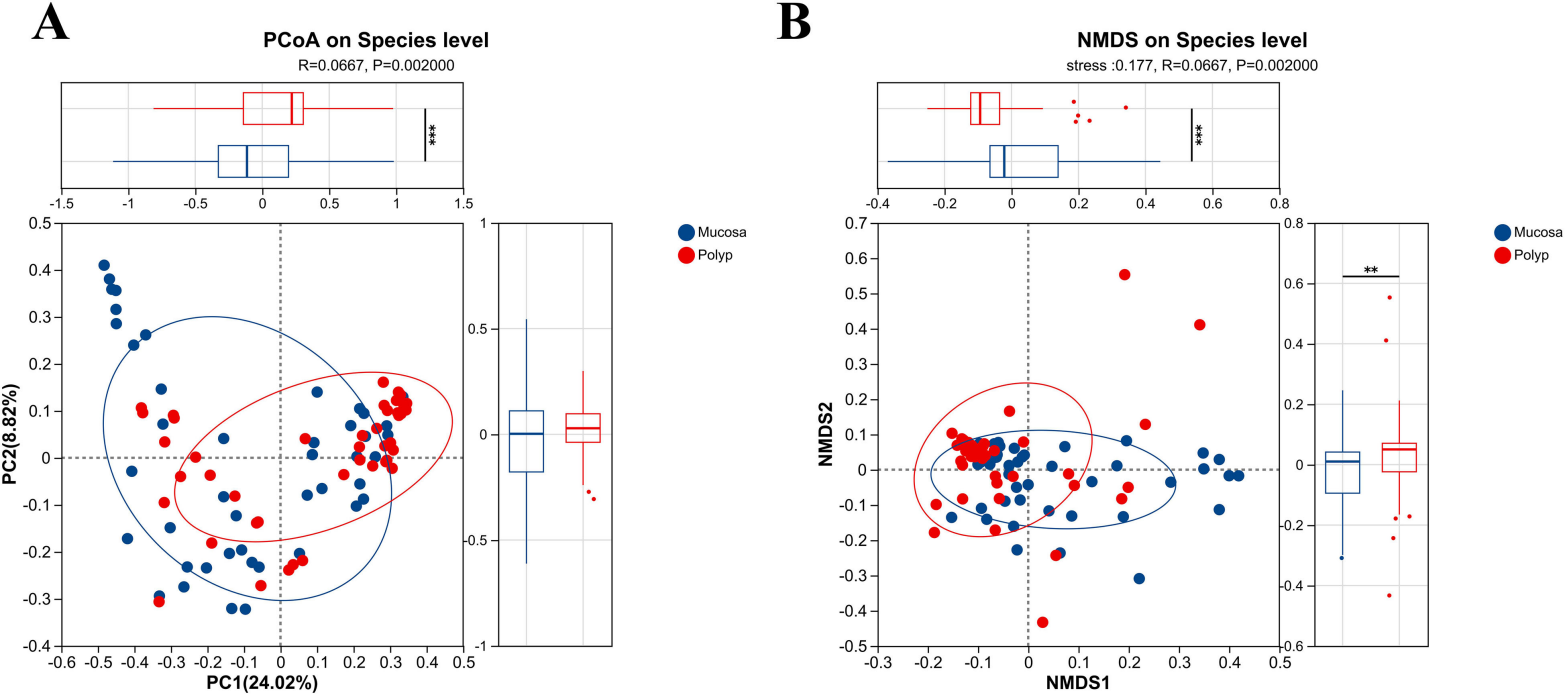
Comparison of beta diversity of the mucosa-associated microbiota between normal mucosa and polyp tissues. **(A)** Principal coordinates analysis (PCoA) and **(B)** non-metric multidimensional scaling (NMDS) plots were generated based on Bray-Curtis distances at the species level. Each point represents an individual sample, colored by group (blue: normal mucosa; red: polyp). The analysis of similarities (ANOSIM) test confirmed a statistically significant separation between the two groups (R = 0.0667, P = 0.002). The PCoA plot shows the percentage of variance explained by each principal coordinate. The stress value for the NMDS ordination is 0.177. ** and *** indicate P < 0.01 and P < 0.001 for intergroup comparisons, respectively.

**Figure 4:**
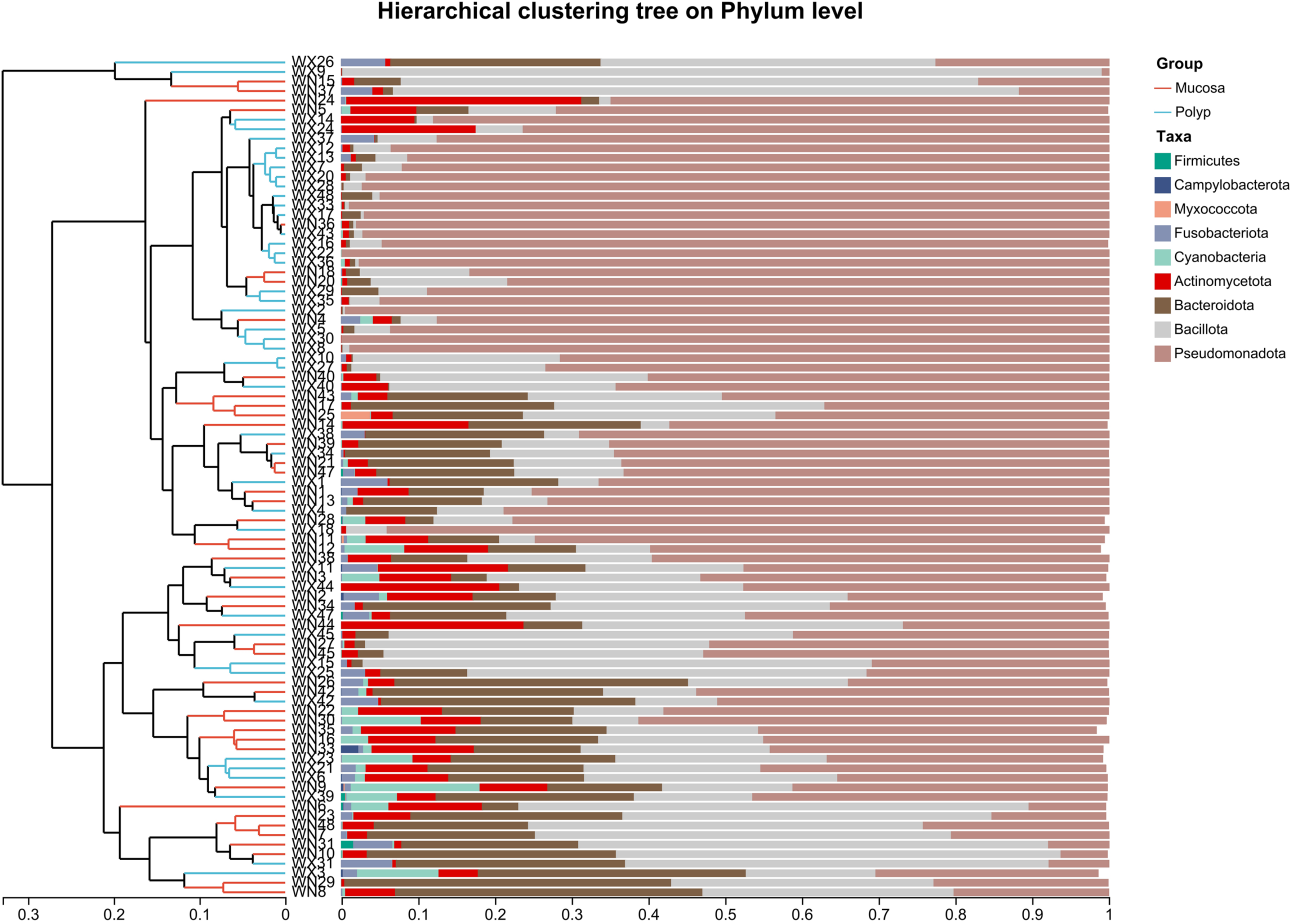
Hierarchical clustering and relative abundance of bacterial phyla in the mucosa-associated microbiota. The dendrogram on the left illustrates the similarity in microbial community structure between samples based on Bray-Curtis distance. The stacked bar chart on the right displays the relative abundance of the dominant bacterial phyla.

PCoA based on Bray–Curtis distances showed significant separation between polyp and normal mucosa groups (R = 0.0667, P = 0.002) (**Figure 3**). NMDS revealed clear intergroup clustering with a stress value of 0.177, indicating reliable multivariate representation. ANOSIM confirmed the statistical significance of these community composition differences (R = 0.0667, P = 0.002). At the phylum level, hierarchical clustering (UPGMA algorithm) using the beta diversity distance matrix yielded a dendrogram with distinct sample clustering, effectively separating most polyp tissues from normal mucosa (**Figure 4**).

### Differential abundance analysis

**Figure 5:**
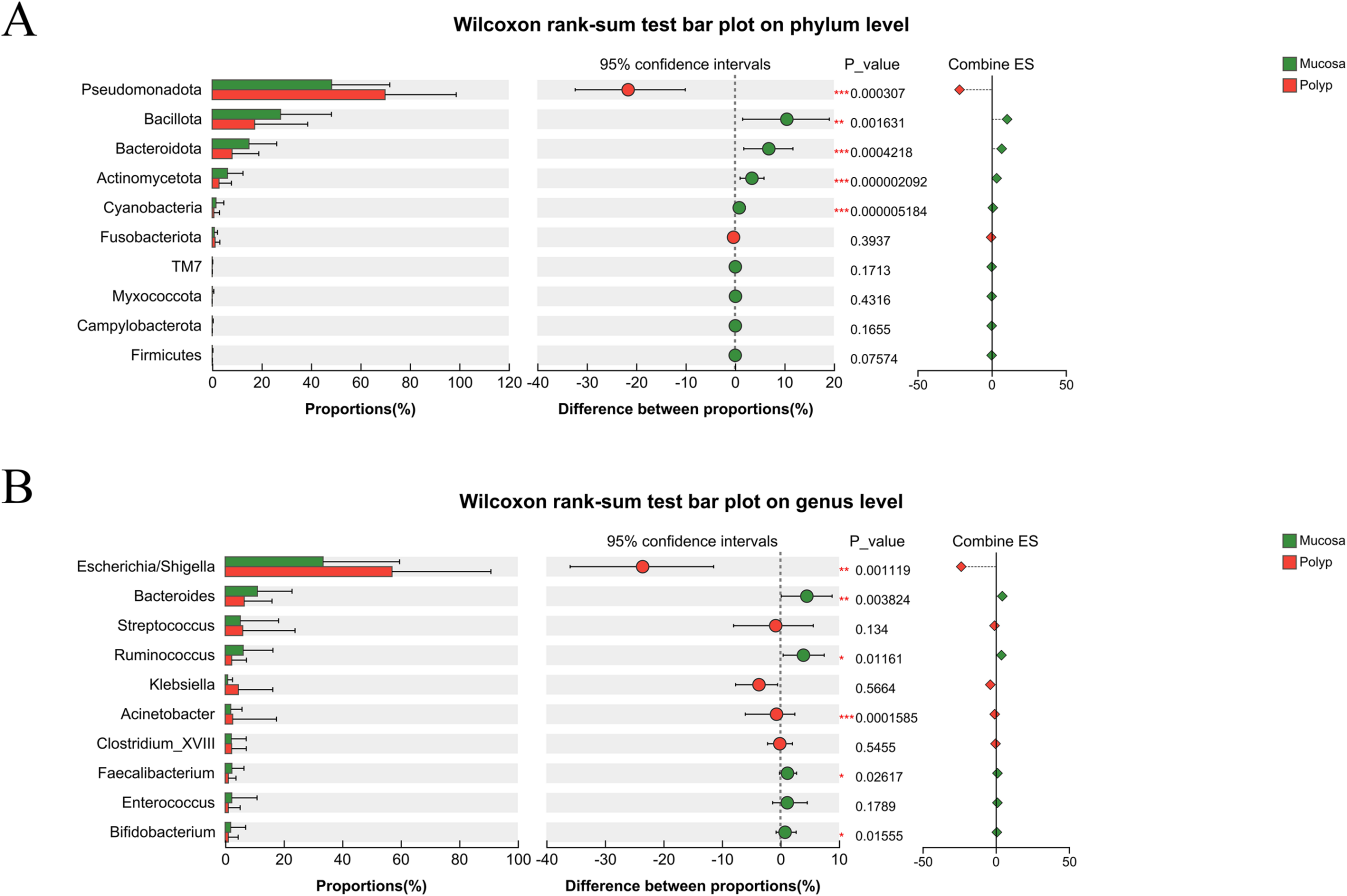
Microbial taxa showing significant differences in abundance between mucosa and polyp groups. Results of the Wilcoxon rank-sum test are displayed for **(A)** phylum-level and **(B)** genus-level taxa. The plot visualizes the proportion of each taxon within the normal mucosa (green) and polyp (red) groups, the difference in proportions (with 95% confidence intervals), P-values, and combined effect sizes. ***, P< 0.001; **, P< 0.01; *,P< 0.05.

Both normal mucosa and PJS polyps were dominated by four major bacterial phyla: *Pseudomonadota*, *Bacillota*, *Bacteroidota*, and *Actinomycetota*. Polyps showed a significant compositional shift, with *Pseudomonadota* relative abundance markedly elevated (70.00% vs. 49.51% in normal mucosa; p < 0.001) and *Bacillota*, *Bacteroidota*, and *Actinomycetota* significantly reduced (**Figure 5A**).

At the genus level, top five taxa in polyps were *Escherichia–Shigella* (56.79%), *Bacteroides* (6.75%), *Streptococcus* (6.37%), *Klebsiella* (4.80%), and *Acinetobacter* (2.88%). Normal mucosa, though also led by *Escherichia–Shigella* (37.61%), had a more diverse community with higher abundances of *Bacteroides* (11.44%), *Ruminococcus* (6.69%), *Streptococcus* (5.09%), and *Faecalibacterium* (2.39%). *Escherichia–Shigella* and *Acinetobacter* were significantly more abundant in polyps, while *Bacteroides*, *Ruminococcus*, *Faecalibacterium*, and *Bifidobacterium* were depleted (**Figure 5B**).

Collectively, these data demonstrate microbial dysbiosis in PJS polyps, characterized by robust enrichment of the pro-inflammatory phylum *Pseudomonadota* (notably *Escherichia–Shigella*) and concurrent loss of commensal bacteria linked to intestinal health.

### Potential microbial biomarkers of PJS polyp

**Figure 6:**
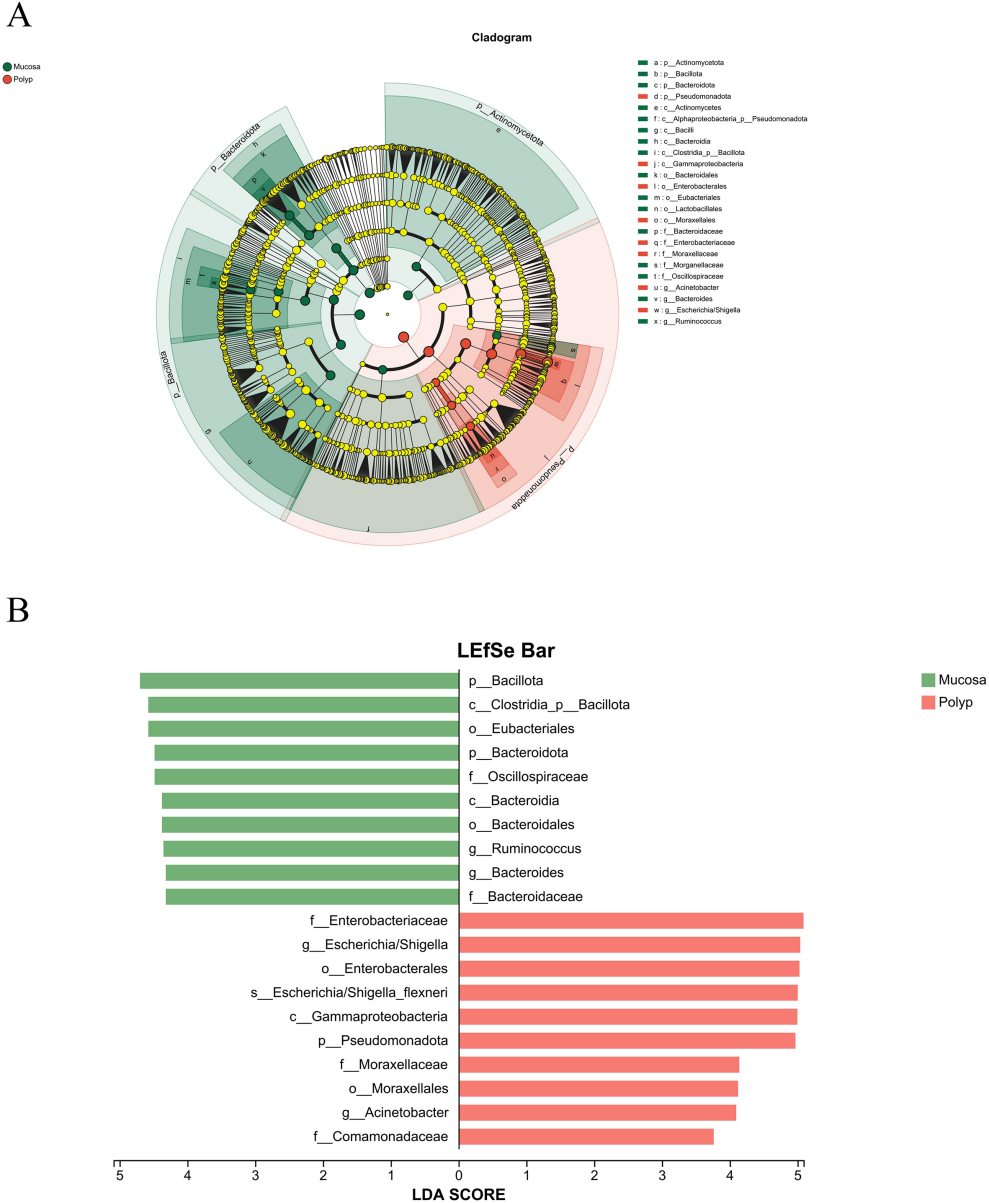
Taxonomic biomarkers distinguishing the mucosal microbiota of normal mucosa and polyp tissues. **(A)** Cladogram illustrating the phylogenetic distribution of bacterial taxa exhibiting significant differences in abundance between the normal mucosa (green) and polyp (red) groups. The circles represent phylogenetic levels from phylum (outermost) to genus (innermost). The diameter of each node is proportional to the relative abundance of the corresponding taxon. **(B)** Histogram of the LDA scores computed from the LEfSe analysis, showing the effect size of each discriminative taxon. Only taxa meeting an LDA significance threshold > 3.5 are displayed.

Linear discriminant analysis effect size (LEfSe) identified phylogenetically differentially enriched bacterial taxa between polyps and normal mucosa (**Figure 6**). In normal mucosa, *Bacillota* and *Bacteroidota* were significantly enriched at the phylum level. At lower taxonomic levels, *Clostridia* and *Bacteroidia* (classes), *Eubacteriales* and *Bacteroidales* (orders), *Oscillospiraceae* and *Bacteroidaceae* (families), and *Ruminococcus* and *Bacteroides* (genera) were also enriched.

In contrast, polyps harbored a distinct set of enriched taxa, with *Pseudomonadota* as the key discriminative phylum. *Gammaproteobacteria* (class), *Enterobacterales* and *Moraxellales* (orders), and *Enterobacteriaceae*, *Moraxellaceae*, and *Comamonadaceae* (families) were significantly enriched. At the genus level, *Escherichia–Shigella* and *Acinetobacter* were overrepresented, while *Shigella flexneri* emerged as the sole differentially abundant species-level biomarker in polyps.

Similarity percentage (SIMPER) analysis determined the contribution of specific taxa to community dissimilarity. The top 10 genera driving these differences are listed in **Table 3**, with *Escherichia–Shigella* showing the highest contribution.

**Table 3:** Top 10 genera contributing to intergroup dissimilarity (SIMPER analysis).

Genus	Contribution (%)
*Escherichia-Shigella*	33.74
*Bacteroides*	8.78
*Streptococcus*	7.17
*Ruminococcus*	5.07
*Klebsiella*	3.90
*Acinetobacter*	3.01
*Clostridium_XVIII*	2.68
*Enterococcus*	2.09
*Faecalibacterium*	2.06

### Microbial dysbiosis index

**Figure 7:**
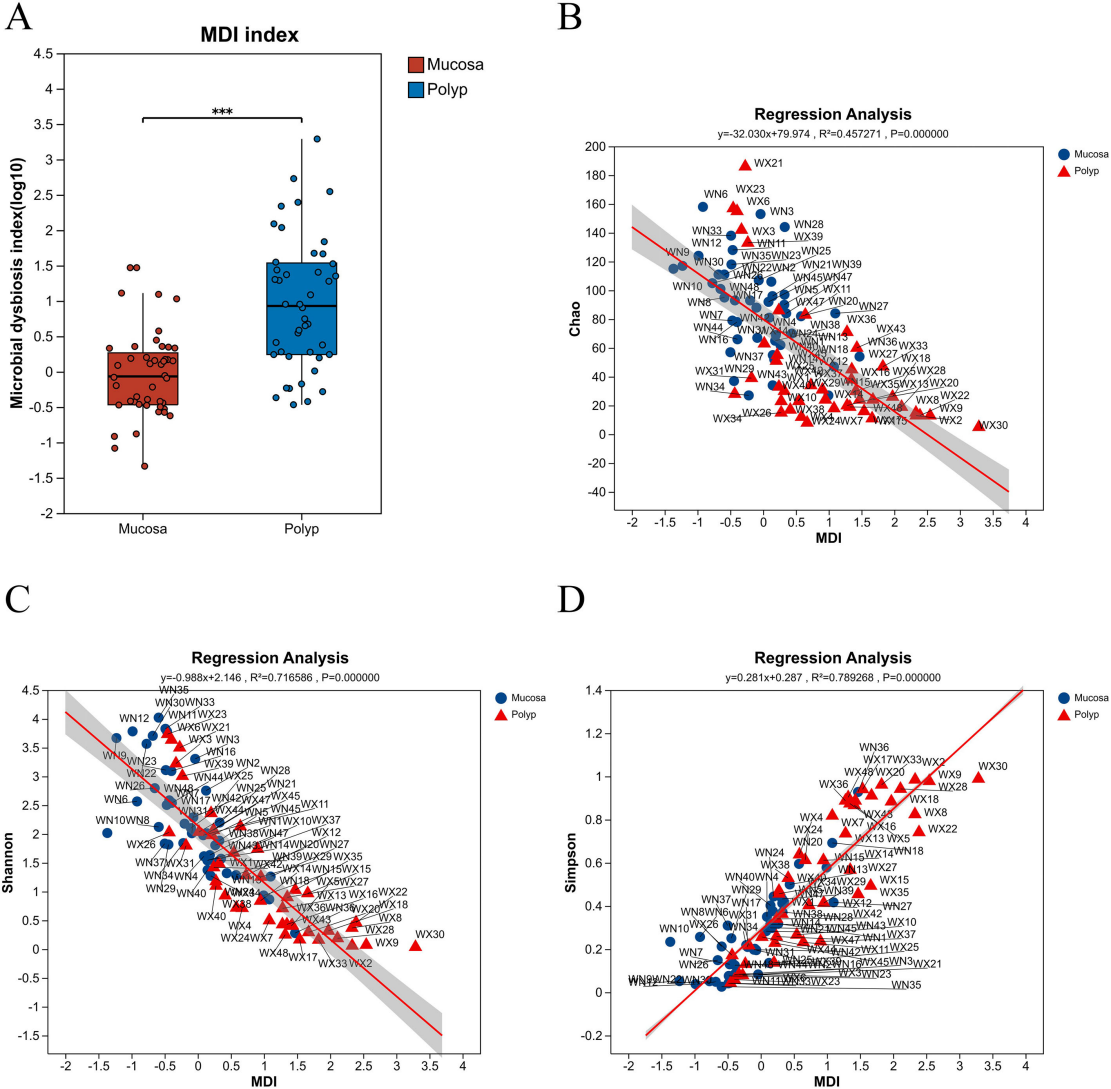
Evaluation of microbial dysbiosis in Peutz-Jeghers syndrome polyps and its correlation with alpha diversity. **(A)** The microbial dysbiosis index (MDI) was significantly elevated in polyp tissues (blue) compared to matched normal mucosa (red) (***P < 0.001). Box plots show the median and interquartile range. **(B–D)** Scatter plots illustrating the significant correlations between the MDI and alpha diversity indices. Each point represents a single sample (red: polyp; blue: mucosa). The solid line represents the linear regression fit.

The microbial dysbiosis index (MDI) was significantly elevated in the polyp group compared with the normal mucosa group (P < 0.001; **Figure 7A**). Furthermore, the MDI exhibited a significant negative correlation with the Chao1 and Shannon indices, and a significant positive correlation with the Simpson’s diversity index (1-D) (**Figures 7B–D**).

### Correlations between gut microbiota composition and clinical parameters

**Figure 8:**
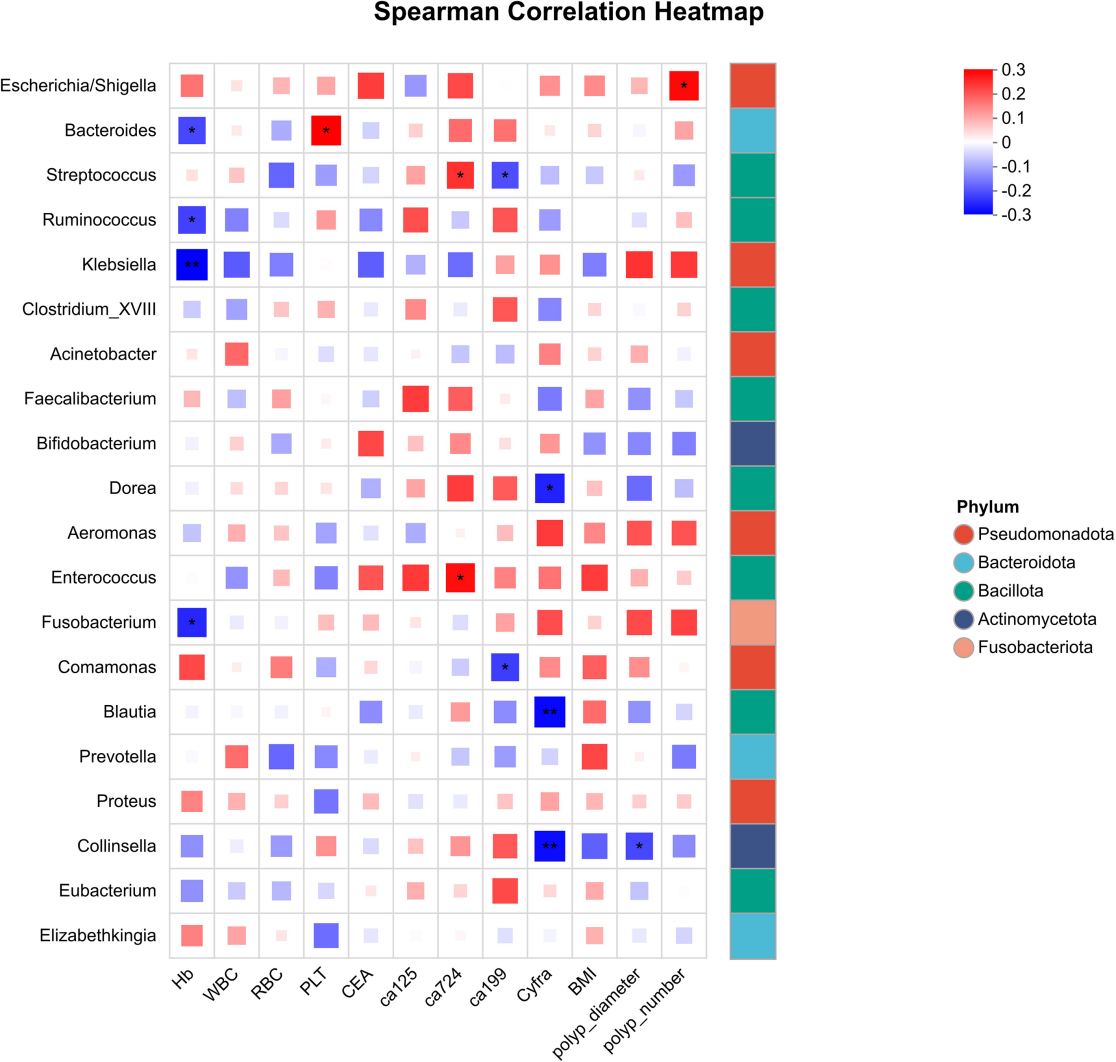
Spearman’s rank correlation heatmap of gut microbiota genera and clinical parameters in PJS patients. Rows denote bacterial genera (color-coded by phylum affiliation; right bar), and columns denote clinical indicators. Each square represents the Spearman correlation coefficient, with size proportional to the absolute correlation value (larger size = stronger association). Color intensity indicates correlation direction and magnitude (range: –0.3 [blue, negative] to 0.3 [red, positive]). **, P < 0.01; *, P < 0.05.

Spearman’s rank correlation analysis was performed to explore relationships between the top 20 bacterial genera and 12 clinical indicators (**Figure 8**). The clinical variables included hemoglobin (Hb), white blood cell count (WBC), red blood cell count (RBC), platelet count (PLT), carcinoembryonic antigen (CEA), carbohydrate antigen 125 (ca125), carbohydrate antigen 72-4 (ca724), carbohydrate antigen 19-9 (ca199), Cyfra 21-1 (Cyfra), body mass index (BMI), largest polyp diameter, and polyp number. Genera belonging to *Pseudomonadota*—including *Escherichia-Shigella*, *Klebsiella*, and *Aeromonas*—showed general positive correlations with polyp burden markers (largest polyp diameter, and polyp number). Specifically, *Escherichia-Shigella* abundance was significantly positively correlated with polyp number (P < 0.05), whereas *Collinsella* abundance showed a significant negative correlation with largest polyp diameter (P < 0.05). Notably, *Enterococcus* abundance correlated positively with all five tumor markers evaluated, with a statistically significant association observed for CA72-4 (P < 0.05). Similarly, *Streptococcus* abundance was significantly positively correlatedignificant association observed for CA72-4 (P with CA72–4 levels (P < 0.05). Additionally, *Klebsiella*, *Bacteroides*, *Ruminococcus*, and *Fusobacterium* exhibited significant negative correlations with hemoglobin (Hb) levels (P < 0.05).

### Microbial biomarker identification via random forest analysis

**Figure 9:**
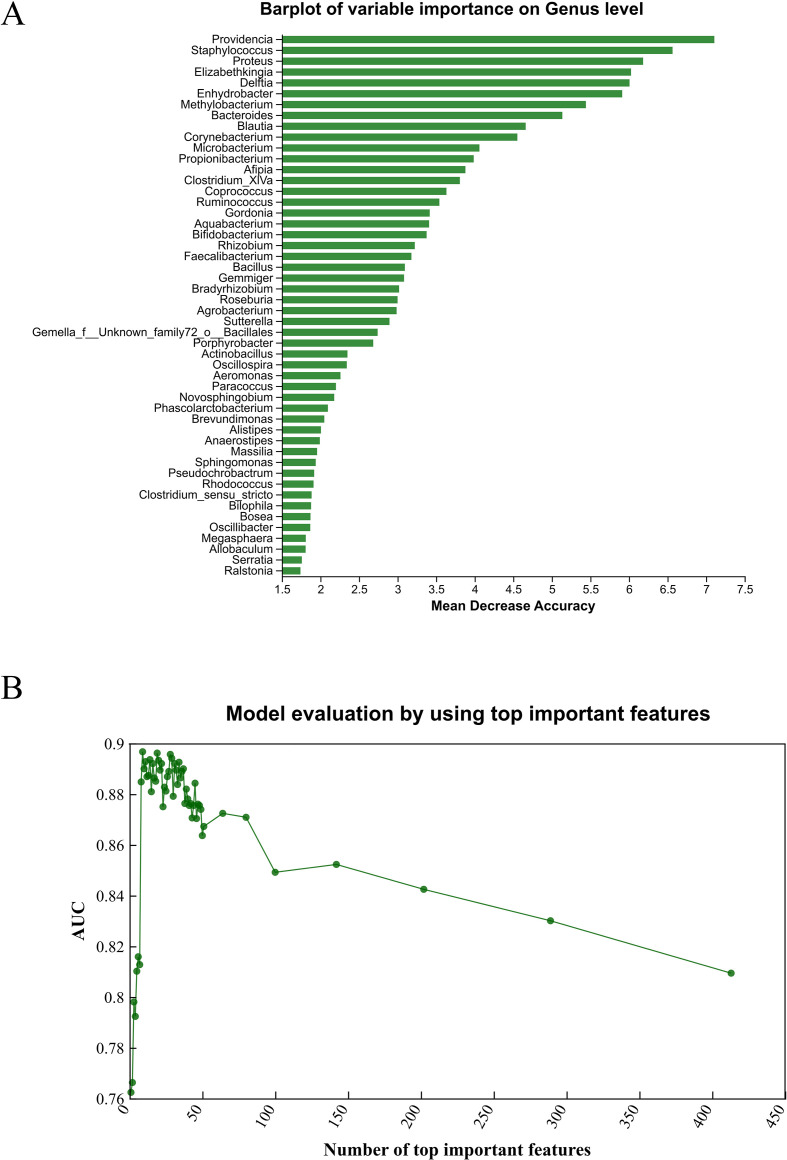
Identification and evaluation of microbial genera distinguishing PJS polyps from normal mucosa via random forest analysis. **(A)** Bar plot depicting mean decrease accuracy of the top 20 most important bacterial genera. This metric represents reduced model predictive performance upon random permutation of a genus’s data; higher values indicate greater classification importance (genera ranked by descending importance). **(B)** Line plot showing model performance (area under the receiver operating characteristic curve, AUC) vs. the number of top-ranked features (genera) included.

To identify a minimal microbial signature distinguishing PJS polyps from normal mucosa, we constructed a random forest model based on genus-level microbiota abundances. Feature importance was evaluated by each taxon’s contribution to classification accuracy, with the top 10 discriminatory genera presented in **Figure 9A**. Model performance was assessed by sequentially incorporating features and calculating the area under the receiver operating characteristic curve (AUC). The model reached optimal discriminative power (AUC = 0.897) upon inclusion of the top nine genera (**Figure 9B**). This signature was defined by increased abundances of *Providencia*, *Staphylococcus*, *Proteus*, *Elizabethkingia*, *Delftia*, *Enhydrobacter*, *Methylobacterium*, *Bacteroides*, and *Blautia*.

### Functional potential of the gut microbiota in PJS polyps

**Figure 10:**
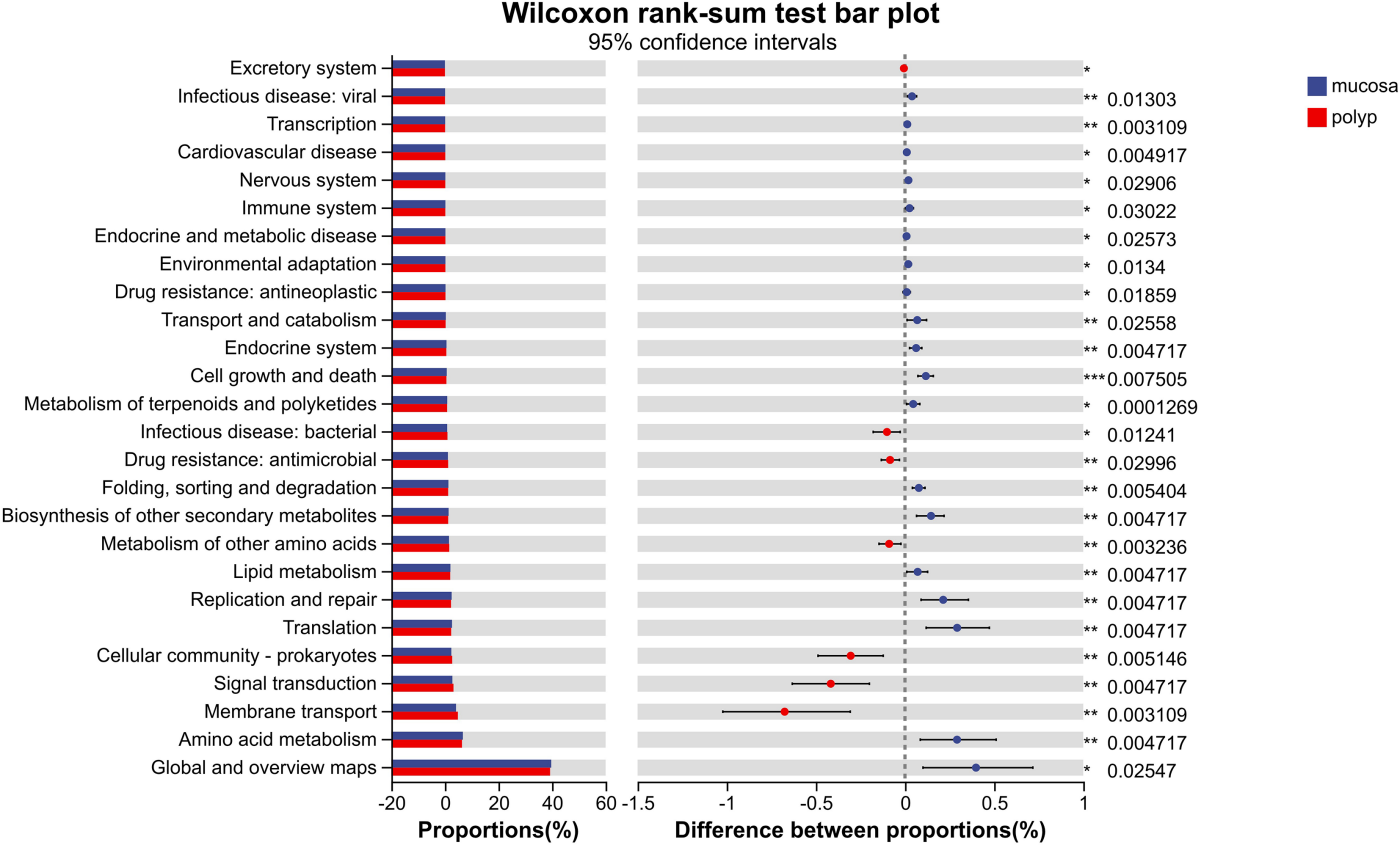
Comparison of predicted microbial functions between PJS polyps and normal mucosa at KEGG Level 2. Left panel: Bar plot showing the mean relative abundance (%) of KEGG Level 2 pathways in each group (normal mucosa, blue; polyps, red). Right panel: Forest plot illustrating intergroup differences with 95% confidence intervals. (***, corrected P-value < 0.001; **, corrected P-value < 0.01; *, corrected P-value < 0.05).

We predicted the functional profiles of the gut microbiota in PJS polyps and paired normal mucosa using PICRUSt2. Twenty-six KEGG Level 2 pathways exhibited significant differential enrichment between the two groups (**Figure 10**; P < 0.05), indicative of distinct predicted functional potential in polyps versus normal mucosa. Relative to normal mucosa, PJS polyps showed marked enrichment in seven pathways: membrane transport, signal transduction, cellular community—prokaryotes, metabolism of other amino acids, antimicrobial drug resistance, bacterial infectious disease, and excretory system. Conversely, 19 pathways were preferentially enriched in normal mucosa, including global and overview maps, amino acid metabolism, translation, replication and repair, lipid metabolism, biosynthesis of other secondary metabolites, folding, sorting and degradation, terpenoid and polyketide metabolism, cell growth and death, endocrine system, transport and catabolism, antineoplastic drug resistance, environmental adaptation, endocrine and metabolic disease, immune system, nervous system, cardiovascular disease, transcription, and viral infectious disease. Collectively, these data suggest that structural microbial dysbiosis in PJS polyps is associated with profound predicted functional reprogramming of the gut microbiome. All functional results are bioinformatic predictions based on 16S rRNA gene data.

## Discussion

Our study, leveraging a rigorously matched polyp-normal mucosa design, identified profound mucosa-associated microbial dysbiosis as a defining feature of the Peutz-Jeghers syndrome (PJS) polyp microenvironment. This dysbiosis—characterized by reduced microbial diversity, a shift toward pro-inflammatory taxa, and predicted functionally reprogrammed metabolic pathways—highlights the microbiota as a potential modulator of PJS polyp pathogenesis, complementing the well-established role of *STK11/LKB1* germline mutations. Consistent with findings in other hereditary colorectal polyposis syndromes (e.g., familial adenomatous polyposis, FAP), microbial dysbiosis is not merely a secondary consequence but a core pathological feature that interacts with germline mutations to influence polyp formation (13).

Our findings align with the limited existing literature on PJS microbiota, which has primarily focused on fecal samples. Two independent studies reported increased *Escherichia-Shigella* and decreased *Faecalibacterium* and *Ruminococcus* in PJS feces (14, 15). Our study extends these findings by demonstrating identical shifts at the mucosal surface, where host-microbe interactions directly occur. We further identified several novel mucosa-specific differential taxa, including *Acinetobacter* (enriched in polyps) and Collinsella (depleted in polyps), which have not been previously reported in PJS fecal studies, highlighting the importance of investigating mucosa-associated communities.

A key finding is the striking ecological imbalance within PJS polyps: alpha diversity is markedly depleted, and beta diversity analyses confirm a distinct microbial community structure that segregates from paired normal mucosa. This separation is not merely a byproduct of inter-individual variation, as our paired-sample design effectively controls for confounding factors such as diet, lifestyle, or genetic background. Subgroup analysis showed no significant differences in diversity between patients with and without prior bowel surgery (all P>0.05), indicating the observed dysbiosis is primarily associated with polyp lesions rather than surgical history. The most prominent taxonomic shift is the dramatic enrichment of *Pseudomonadota*, driven primarily by the expansion of *Escherichia–Shigella*—a pattern eerily reminiscent of microbial signatures reported in sporadic colorectal cancer (CRC) (13). This convergence suggests a shared microbial “pro-neoplastic” pathway transcending initial genetic defects.

We propose a two-stage ‘genetics-first, microbiota-amplified’ model for PJS polypogenesis, consistent with the bacterial driver-passenger theory (16). Germline *STK11/LKB1* mutations act as the initial genetic driver, disrupting epithelial polarity, impairing immune surveillance, and creating a permissive microenvironment for pro-inflammatory pathobionts such as *Escherichia-Shigella*. These pathobionts then function as passenger-drivers, producing genotoxins (e.g., colibactin) and inducing chronic inflammation that damages epithelial DNA and accelerates polyp growth. This model is supported by findings in familial adenomatous polyposis (FAP), where *APC* mutations precede and promote expansion of tumorigenic mucosal bacteria (17). While plausible, direct mechanistic studies are needed to confirm these interactions.

Members of *Escherichia–Shigella* (e.g., pathogenic *E. coli*) are well-documented to be linked to intestinal inflammation and genomic instability via genotoxin production (e.g., colibactin) and modulation of host immune responses (18); this is analogous to observations in FAP, where mucosa-associated *E. coli* (pks+ strain) and *Bacteroides fragilis* form biofilms and drive polypogenesis through similar mechanisms (17). Their positive correlation with polyp number in our cohort raises the intriguing possibility that these pathobionts exacerbate disease progression by sustaining chronic mucosal irritation and impairing DNA repair—processes that may synergize with *STK11/LKB1* deficiency to influence polyp growth, as seen in *STK11*-deficient mouse models colonized with pro-neoplastic bacteria (16, 17).

Concurrently, the depletion of beneficial commensals such as *Faecalibacterium* and *Ruminococcus*—key producers of butyrate, a short-chain fatty acid with anti-inflammatory and tumor-suppressive properties—likely further disrupts gut homeostasis by reducing microbial-mediated immune surveillance and enhancing the pro-inflammatory milieu. Butyrate exerts anti-inflammatory and anti-tumor effects through three key mechanisms (1): Inhibition of histone deacetylases to suppress proliferation and induce apoptosis (2); Activation of GPR41/GPR43 to enhance barrier function (3); Promotion of regulatory T cell differentiation and inhibition of pro-inflammatory cytokines (4). Preclinical studies confirm reduced butyrate production accelerates CRC progression (8), probiotic supplementation reduces polyp burden in ApcMin/+ mice (19), and *STK11*-deficient mice exhibit impaired butyrate metabolism (20). The significant elevation of the Microbial Dysbiosis Index (MDI) in polyps, and its strong correlations with alpha diversity indices, quantifies this ecological disturbance and reinforces its biological relevance.

Correlations between microbial taxa and clinical parameters provide additional insights into host-microbe interactions in PJS. The negative association between *Collinsella* abundance and largest polyp diameter hints at a potential protective role for this genus, though its functional mechanisms—whether via metabolite production, immune modulation, or competition with pathobionts—warrant further investigation.

Of particular interest is the positive correlation of *Enterococcus* with all five evaluated tumor markers, including a significant association with CA72-4. This finding suggests that *Enterococcus* may serve as an adjunct biomarker for malignant transformation risk in PJS. CA72–4 is a widely used gastrointestinal tumor marker but has limited specificity for early cancer detection. Combining *Enterococcus* abundance with CA72–4 could improve the positive predictive value for identifying PJS patients at highest risk of developing gastrointestinal malignancies. Future prospective studies should validate whether fecal *Enterococcus* levels can predict incident cancer in PJS cohorts. *Enterococcus* species have been correlated with CRC and liver cancer pathogenesis through their ability to trigger pro-inflammatory signaling cascades and promote cellular proliferation (21, 22), suggesting they may contribute to the elevated cancer risk in PJS by modulating systemic oncogenic pathways. Similarly, the positive correlation between *Streptococcus* and CA72–4 levels aligns with emerging evidence that specific oral and gut bacteria can serve as non-invasive biomarkers for neoplastic risk (23).

Additionally, the negative correlations between *Klebsiella*, *Bacteroides*, *Ruminococcus*, *Fusobacterium* and hemoglobin levels suggest that microbial dysbiosis may contribute to anemia—a common comorbidity in PJS—potentially via dysregulating nutrient absorption or sustaining low-grade inflammation that impairs erythropoiesis. This aligns with reports that gut microbiota dysbiosis disrupts iron metabolism and erythropoietin responsiveness in hereditary polyposis (13), and that specific microbial taxa (e.g., *Fusobacterium*) are linked to systemic inflammatory markers that exacerbate anemia (24). Notably, microbial signatures have also been proposed as non-invasive biomarkers for neoplastic risk in other polyposis syndromes, supporting our observation that *Enterococcus* and *Streptococcus* correlate with tumor markers (25, 26).

PICRUSt2-predicted functional profiles reveal predicted metabolic reprogramming of the polyp-associated microbiota. Enrichment of pathways linked to membrane transport, signal transduction, and antimicrobial drug resistance reflects adaptive strategies of the Pseudomonadota-dominated community, likely enabling its survival and expansion in the pathological polyp microenvironment. Conversely, depletion of pathways involved in amino acid metabolism, translation, and biosynthesis mirrors the loss of metabolic functions typically conferred by beneficial commensals. Most notably, the predicted reduction in immune system-related pathways raises the hypothesis that the dysbiotic microbiota contributes to the immunosuppressive niche characteristic of *STK11*-deficient tissues (27). Germline STK11 deficiency in immune cells is known to impair anti-tumor immunity and promote polyposis (20); our data suggest the microbiota may act as a critical intermediary in this process, amplifying immune tolerance and creating a permissive environment for polyp initiation and growth. Consistent with spatial profiling studies of intratumoral microbiota, pro-inflammatory taxa (e.g., *Escherichia–Shigella*) likely accumulate in immunosuppressive microniches characterized by reduced T cell infiltration, a phenomenon also observed in FAP where resident memory T cells and γδ T cells are depleted, weakening microbial surveillance (25). This “genetics-microbiota-immunity” axis represents a novel framework for understanding PJS pathogenesis, paralleling similar axes described in FAP and Lynch syndrome (13, 19). Importantly, these functional inferences require validation through shotgun metagenomics or targeted metabolomics to confirm actual microbial metabolic activity in PJS lesions.

From a translational perspective, our findings hold two key implications. First, the random forest model identified a panel of nine microbial genera that discriminates polyps from normal mucosa with high accuracy (AUC = 0.897). However, its clinical applicability is currently limited by several factors (1): it is a tissue-based biomarker requiring invasive enteroscopy for sampling (2); it has only been validated to distinguish polyps from adjacent normal mucosa within PJS patients, not to distinguish PJS patients from healthy individuals or patients with other polyposis syndromes (3); it has not been evaluated for its ability to predict polyp growth, recurrence, or malignant transformation; and (4) it lacks external validation in independent cohorts. Future studies should investigate whether this signature can be detected in non-invasively collected fecal samples and validate its performance in larger, multi-center PJS cohorts. This microbial signature offers promise as a tissue-based biomarker for assessing disease activity or stratifying cancer risk in PJS patients, complementing existing clinical and genetic tools. Building on this signature, future studies could develop a PJS Microbial Activity Score (PJS-MAS) and integrate it with clinical parameters (age, polyp burden, family history of cancer) and tumor marker levels to construct a multi-variable cancer risk stratification model. Second, the microbiota represents a malleable target for therapeutic intervention. While *STK11/LKB1* mutations are irreversible, targeted strategies—such as selective depletion of pathobionts (e.g., *Escherichia–Shigella*) via narrow-spectrum antimicrobials or phage therapy, or supplementation with butyrate-producing probiotics (e.g., *Faecalibacterium prausnitzii*, *Ruminococcus bromii*)—could restore microbial homeostasis and potentially suppress polyp progression. Preclinical studies in FAP have demonstrated that probiotic supplementation reduces intestinal inflammation and polyp burden (19), and phage therapy targeting *Escherichia–Shigella* effectively shrinks polyps in patient-derived models (13, 17). Additionally, non-steroidal anti-inflammatory drugs (NSAIDs) may synergize with microbiota modulation to enhance barrier function and reduce pro-inflammatory taxa (24).

### Limitations

Several limitations of our study merit consideration. First, our study included a relatively small cohort of 44 PJS patients, which reflects the rarity of this disease. Larger multi-center cohorts are needed to confirm our findings and identify less abundant microbial signatures associated with PJS pathogenesis. Second, although functional predictions derived from PICRUSt2 provide valuable insights, they remain inferential and require validation through shotgun metagenomic sequencing and targeted metabolomic analyses. These complementary approaches would confirm the actual metabolic activity of the polyp-associated microbiota and the *in situ* production of key metabolites (e.g., butyrate) in PJS lesions. Third, our cross-sectional study design only establishes associations between microbial dysbiosis and PJS polyposis, not causal relationships. To definitively delineate whether dysbiosis influences polyp initiation or progression, future studies should include longitudinal follow-up to track dynamic changes in the microbiota alongside polyp growth trajectories, as well as mechanistic experiments using *STK11*-deficient animal models colonized with microbiota isolated from PJS patients. Fourth, polyp burden was quantified using conventional endoscopic metrics (i.e., polyp number and maximum diameter), which are inherently subjective owing to inter-operator variability in endoscopic technique, luminal distention, and lesion measurement. Future investigations should adopt more objective assessments, such as total polyp volume calculated via 3D endoscopic reconstruction or composite severity scores integrating radiographic imaging (e.g., computed tomography colonography) with endoscopic findings. Fifth, our study is limited to genetically confirmed PJS patients from a single center, consisting exclusively of Han Chinese patients, limiting the generalizability of our findings due to known influences of ethnicity and lifestyle habits on gut microbiota composition. Microbial signatures in polyposis syndromes are known to exhibit population-specific variations (26), highlighting the need for validation in larger, geographically diverse multi-center cohorts. Sixth, our study only compared polyp and paired normal mucosa from PJS patients and did not include a healthy control group. Therefore, we cannot determine whether the observed microbial alterations in PJS normal mucosa are already abnormal compared to the general population, or whether the polyp-associated dysbiosis is unique to PJS or shared with other hereditary polyposis syndromes. Seventh, bowel preparation prior to DBE may transiently alter microbiota, though mucosa-associated communities are relatively resistant and our paired design minimizes this impact. Eighth, while we implemented a comprehensive negative control system and rigorous contamination filtering pipeline, we acknowledge that low-biomass mucosal microbiome studies remain inherently susceptible to trace contamination. Future studies should further optimize contamination control strategies, including the use of multiple independent DNA extraction kits and sequencing platforms to validate findings. Furthermore, future studies should integrate metabolomic analyses to confirm functional metabolic shifts (e.g., reduced butyrate production) predicted by PICRUSt2, a strategy recently validated in cross-syndrome polyp microbiota research (26).

## Conclusion

In summary, our study identified marked mucosal microbial dysbiosis as a key feature of Peutz-Jeghers syndrome (PJS) polyps, characterized by reduced microbial diversity, enriched pro-inflammatory *Pseudomonadota* (notably *Escherichia–Shigella*), depleted beneficial commensals, and profound predicted functional reprogramming. We identified a 9-genera microbial signature with potential biomaker value and proposed a “genetics-first, microbiota-amplified” model for PJS polypogenesis. These findings provide novel insights into PJS pathogenesis and suggest microbiota-modulating strategies as promising adjunctive therapies.

## Data Availability

The raw 16S rRNA gene sequencing reads generated in this study are available in the NCBI Sequence Read Archive (SRA) under BioProject accession number PRJNA1402014.
